# A wheat canopy albedo high-throughput phenotyping method and its relationship with canopy architecture and leaf properties

**DOI:** 10.3389/fpls.2026.1769886

**Published:** 2026-02-25

**Authors:** M. F. Ruz-Ruiz, Jose A. Jimenez-Berni, Richard Trethowan, Helen Bramley

**Affiliations:** 1 Institute for Sustainable Agriculture, Spanish National Research Council (IAS-CSIC), Cordoba, Spain; 2 School of Life and Environmental Sciences, Plant Breeding Institute, Sydney Institute of Agriculture, The University of Sydney, Narrabri, NSW, Australia; 3 School of Life and Environmental Sciences, Plant Breeding Institute, Sydney Institute of Agriculture, The University of Sydney, Cobbitty, NSW, Australia

**Keywords:** canopy albedo, heat stress, hyperspectral, phenotyping, wheat

## Abstract

Heat stress damage leads to yield penalties in many wheat-growing areas. Climate change models predict warmer scenarios and more frequent heat shocks. Consequently, wheat breeders need to develop more productive varieties for warm conditions, and therefore, the identification of heat-tolerance traits is needed. Albedo is an integrative trait of the optical properties of the canopy defined as the ratio of reflected light to total light received. High albedos in warm conditions may help reduce damaging radiation. Despite its potential relevance for heat avoidance, albedo has been little explored in wheat breeding. In this work, a selection of 30 wheat (*Triticum aestivum* L.) genotypes of diverse origin were sown at two sowing dates in Australia (NSW) in 2018 and 2019. A high-throughput phenotyping method based on spectroradiometer measurements [Analytical Spectral Devices (ASD)] to measure canopy albedo was developed to explore its relationship with temperature and other heat tolerance-related traits. ASD albedo was validated via continuous albedometer measurements on a subset of genotypes. Data were captured at flowering (one of the most critical periods for heat-related damage). Genotypic differences for albedo were found in most environments. However, genotypic effects were most noticeable at noon in optimally sown materials (H^2^ 0.71–0.86). Albedo was directly related to canopy architecture and light interception (r = 0.74) and varied depending on genotype and genotype by environment interaction. Air temperature in the canopy profile and canopy temperature (CT) were also monitored continuously in a subset of genotypes to explore the relationship between albedo and canopy micrometeorology. Canopies with higher albedos had larger air temperature differences across the canopy profile at the flowering stage (r = 0.48). However, canopy temperature was not related to albedo, even though it was strongly correlated (r = 0.99) with air temperature around the spike. Overall, these results indicate that canopy architecture is the primary influence on albedo under warm conditions. Although higher albedo was not associated with lower canopy temperature, its influence on canopy micrometeorology suggests that albedo may contribute to heat avoidance and could therefore be considered an additive trait for phenotyping and breeding for environments under high temperatures.

## 1 Introduction

The ability of a crop to capture and convert light energy and CO_2_ to biomass (i.e., its radiation use efficiency) influences growth and yield. Wheat (*Triticum aestivum* L.) genotypes differ in their canopy architecture, so light distribution and photosynthesis vary within the canopy profile (Richards et al., 2019; Salter et al., 2020; Ponce de León and Bailey, 2024). It is believed that optimising canopy structures to enhance radiation fluxes between the canopy and atmosphere, and stabilising vertical light distribution within the canopy, could increase yield (Salter et al., 2020). However, in high irradiance situations, photo-oxidative damage and canopy overheating can occur (Li et al., 2009). Canopy structure traits are therefore needed to counteract the injury induced from high light interception and avoid high canopy temperatures, especially in warm environments. Canopy albedo may be a suitable trait, as it is the ratio of intercepted solar radiation that is reflected out of the canopy to the atmosphere (Drewry et al., 2014; Zhang et al., 2022a; Lei et al., 2024). Higher albedo reduces the radiation absorbed by plants, so alterations to canopy traits to optimise albedo could help reduce overheating and mitigate injury to sustain yield increases in warm environments.

Canopy albedo changes during the growing season for wheat crops, reaching a maximum around heading, decreasing towards maturity, and increasing again at senescence (Mo and Suxia, 1997; Zhang et al., 2013; Lunagaria, 2021). Canopy albedo also changes diurnally, with atmospheric and surface conditions causing asymmetry in the daily expression of albedo (Dexter et al., 2004; Nagai, 2002; Zhang et al., 2013). Variances in albedo are related to changes in canopy structure and, therefore, properties such as leaf angles, leaf rolling, canopy cover (leaf area index), and also leaf optical properties (Bsaibes et al., 2009; Drewry et al., 2014). These traits not only vary between wheat genotypes but also vary under high-temperature conditions. However, the relationship between heat tolerance (here defined as the ability to yield well under high temperatures) or heat avoidance (here defined as cooler canopies under high temperatures) and albedo has been little studied. Şerban et al. (2012) compared albedo at midday in different wheat genotypes and found different responses before and after flowering, while Idso et al. (1977) used albedo measurements to validate a yield predictor formula under heat stress. Also, considering albedo at large-scale canopies, Ridgwell et al. (2009) concluded that albedo can help mitigate surface temperature and estimated that increasing albedo by 20% can decrease surface air temperature by 1°C.

Albedo is usually defined as the ratio of two measurements using pyranometers: one directed at the sky measuring incoming solar irradiance and the second at the surface of interest measuring canopy reflected irradiance. These measurements are traditionally obtained at a stationary point or using remote sensing applications (Gutman et al., 1989; Bsaibes et al., 2009; Zhu et al., 2024). However, there is very little evidence of the application of quantitative measurements of albedo in plant phenotyping or breeding. High-throughput phenotyping of albedo could help screen wheat genotypes for heat tolerance. In fact, canopy hyperspectral measurements have been used to predict yield (Wang et al., 2003; Bowman et al., 2015) and other traits (Silva-Perez et al., 2018; Furbank et al., 2021).

Canopy albedo is related to specific leaf properties and canopy architecture parameters (Richards et al., 2019; Ponce de León and Bailey, 2024). These attributes can be manipulated through agronomic practice and plant breeding to achieve cooler canopies, thus reducing the exposure to heat stress (Richards et al., 2014; Kiranakumara et al., 2024). For example, cuticular wax is the hydrophobic interface between plants and the environment, protecting them from biotic and abiotic stresses. When the wax deposits form filaments extending from the plant surface, the leaf shows a “bloom” or greyish colour, which is called glaucousness (Esau, 1960). Many reviews have stated that glaucousness reduces canopy temperature and is thus a valuable breeding target for improving yield in hot environments (Hunt et al., 2018), despite often contradictory evidence (Bennett et al., 2012). The seminal paper of Richards et al. (1986) demonstrated a relationship between leaf glaucousness and temperature in wheat. Considering that the abaxial side of a wheat leaf usually expresses more glaucousness and the level of glaucousness often increases under high temperatures (Bramley et al., 2022), Richards et al. (1986) suggested that it may protect the emerging spike as flag leaves would be vertical, thus exposing the abaxial glaucous side while radiation and temperature are increasing during this growth stage. After the spike has emerged, flag leaves also tend to roll in hot and dry conditions, exposing the abaxial side while protecting the adaxial side, which has higher stomatal densities and transpiration rates (Ranawana et al., 2021).

Leaf distribution is also an important factor affecting the canopy environment (Pearcy et al., 1989; Ross, 2012; Costes et al., 2013). The light environment within the canopy and radiation exchange is strongly influenced by the canopy structure, and thus, light interception is an important feature for characterising the canopy (Pearcy et al., 1989). Modern wheat cultivars already tend to have smaller, more erect leaves, especially in the upper canopy, which allow light to penetrate (Long et al., 2006), thereby increasing photosynthesis and avoiding light saturation in the upper leaves. In hot/dry environments and high solar exposure scenarios, plants tend to have smaller leaves that roll, thereby capturing less radiation and reducing overheating (Kadioglu and Terzi, 2007; Balota et al., 2008; Ali et al., 2022). Planophile canopies have greater reflectance than erectophile types in the near-infrared region, whereas erectophile canopies have higher reflectance in the visible waveband (Yanli et al., 2007). However, the role of leaf morphology in relation to canopy architecture and its effects on the capacity to withstand high temperatures remains unclear.

Canopy height also modulates the canopy structure. However, the relationship between canopy height and canopy temperature is still not clear. Mason and Singh (2014) found no relationship between these two variables, while Li et al. (2019) showed that root depth was the main driver of canopy temperature and that deeper roots were associated with taller plants.

When wheat is under heat stress, every degree over the optimum temperature (between 5°C and 28°C in flowering) reduces yield (Asseng et al., 2011). The canopy microclimate has been studied widely (Penman and Long, 1960; Waggoner and Reifsnyder, 1968; Maitani et al., 1995; Mo and Beven, 2004), including the energy fluxes occurring between canopy elements and architecture. Cleugh (2002) studied how growth and evaporation were impacted by incorporating micrometeorology variables into crop simulation models. However, micrometeorology in the canopy under heat stress has not been studied. Implementing these variables can help understand the canopy environment and may assist in the identification of interesting traits.

Canopy temperature (CT) and canopy temperature depression (CTD) are used directly as screening variables for heat tolerance (Reynolds et al., 1994; Farooq et al., 2009). CTD and CT depend on canopy architecture, transpiration rates, and wheat genotype (Huang et al., 2024). Many studies have reported a positive correlation between CTD and yield in wheat (Reynolds et al., 1994; Amani et al., 1996; Ayeneh et al., 2002).

In this paper, we tested the hypothesis that albedo is an integrative trait of canopy architecture by exposing a diverse set of wheat genotypes with varying heat tolerance to high temperatures in the field during two sowing dates. We developed and validated a high-throughput method for estimating broadband canopy albedo using measurements of spectral reflectance obtained from a spectroradiometer in the full range of the shortwave radiation (350–2,500 nm). We then explored the relationship between albedo and leaf and canopy properties, including canopy temperature and air temperature in the profile at the flowering stage of development, when plants are most susceptible to heat stress (Farooq et al., 2011). Canopy albedo is therefore considered a heat-avoidance trait since it modulates the incoming radiation and the microclimate.

## 2 Materials and methods

### 2.1 Field experiments

Thirty wheat genotypes with contrasting yield performance under high temperatures at flowering (where daily air temperatures of ≥28°C exceed the optimum for reproductive development) were selected from a diverse set of breeding lines and Australian cultivars (details included in **Supplementary Material**; **Supplementary Table S1**). The wheat genotypes were evaluated for albedo, morphological, and physiological properties in 2018 and 2019 in field trials located near Narrabri (lat. 30.3324°S, long. 149.7812°E) at the IA Watson Grains Research Centre, NSW, Australia. Two sowing dates were established—optimal (TOS 1) and late (TOS 2)—so that genotypes were exposed to high temperatures at critical growth stages. TOS 1 occurred around mid-May (15/05/2018 and 17/05/2019), and TOS 2 occurred 8 weeks later (16/07/2018 and 15/07/2019).

Experiments were arranged in randomised complete block designs of four replicate plots at each time of sowing and irrigated using a lateral irrigator when needed to avoid the confounding effects of water stress. Soil water content was monitored using neutron probes uniformly distributed throughout the experiment, and these were checked fortnightly. Trials were sown using a five-row precision planter operated by Australian Grain Technologies (AGT) in plots of 12 m^2^ at a plant density of 100 plants m^−2^. Narrabri soils are predominantly chocolate vertosols according to the Australian Soil Classification and have a plant available water capacity of 190 mm to a depth of 1.2 m (Baird and Lonergan, 2018). Chemical spray applications and soil treatments followed the common practices of the area.

### 2.2 Canopy albedo measurements

Four genotypes were selected to install albedometers based on contrasting canopy traits assessed from a field experiment in 2017 and breeding values from The University of Sydney’s plant breeding program (project US0081 supported by the Grains Research and Development Corporation). Measurements were conducted in TOS 1 and TOS 2 trials in 2018 and only in TOS 1 in 2019. Broadband canopy albedo at the flowering stage was monitored using albedometers (SRA01 Hukseflux, Delft, Netherlands) installed for 24 h on selected genotypes in three blocks randomly chosen from the four available, with one block assessed per day. Flowering was defined as when anthers were visible on 50% of the spikes in the plot at Zadoks stage 65 (Zadoks et al., 1974). The albedometers consisted of two identical pyranometers that measured incoming hemispherical solar radiation and reflected solar radiation (285 to 3,000 × 10^−9^ m), one downfacing the canopy and one upfacing the atmosphere, mounted on a pole with a bracket so that the sensor could reach the middle of the plot at 0.50-m height above the canopy. The albedometers sent data to the cloud using LoRaWAN technology through The Things Network (https://www.thethingsnetwork.org/) using Pycom (UK) boards based on dataloggers connected to a multichannel analog-to-digital converter.

### 2.3 Canopy spectral reflectance measurements

One-off canopy reflectance measurements were captured at flowering on all genotypes three times a day (early morning, solar noon, and mid-afternoon). These measurements were conducted on one block per day, coinciding with the block where albedometers were installed. Unlike the albedometer measurements, reflectance was measured on all genotypes within the block, so that the data collection was coincident in time with the albedo monitoring. A spectroradiometer [Analytical Spectral Devices (ASD) FieldSpec^®^ 3, ASD Inc., Boulder, CO, USA] with a pistol grip to hold the fibre optic cable was used, leaving a 25° view angle with the horizontal. The pistol was held facing the canopy and moved in a zigzag motion along the long side of the plot, avoiding the edges. Four radiance spectra per plot were recorded, where each was configured to an average of 25 spectra. To calculate canopy reflectance, four radiance spectra of a calibrated ~99% Lambertian reference panel (Spectralon^®^, LabSphere, North Sutton, USA) were recorded approximately every 15 minutes, where every spectrum was the average of 100 spectra. The white reference panel was installed horizontally on a camera tripod. Dark current spectra were recorded automatically and comprised the average of 100 spectra. Reflectance was calculated as the ratio of the canopy radiance and the time-interpolated radiance from the reference panel. Albedo per plot was obtained by calculating the average of the full-range spectra, with the following considerations: i) reflectance measurement was directly convertible into the surface albedo value, assuming a Lambertian behaviour of the surface; ii) wavelength bands where water was absorbed (1,350–1,420, 1,800–1,950, and 2,400–2,501 nm) were excluded to minimise noise; and iii) a different spectroradiometer unit, but the same model, was used for measurements in TOS 2 in 2018 due to the original being unavailable for loan.

### 2.4 Traits in the field

The leaf properties of glaucousness, leaf width, leaf angle, and leaf rolling were evaluated at flowering for all genotypes, whereas leaf length was only assessed on a subset of varieties. Canopy architecture-related traits were evaluated, such as canopy height and normalised difference vegetation index (NDVI) on the full set of genotypes, whereas light interception was monitored throughout the day on the same subset of genotypes monitored for daily albedo.

#### 2.4.1 Glaucousness

**Figure 1:**
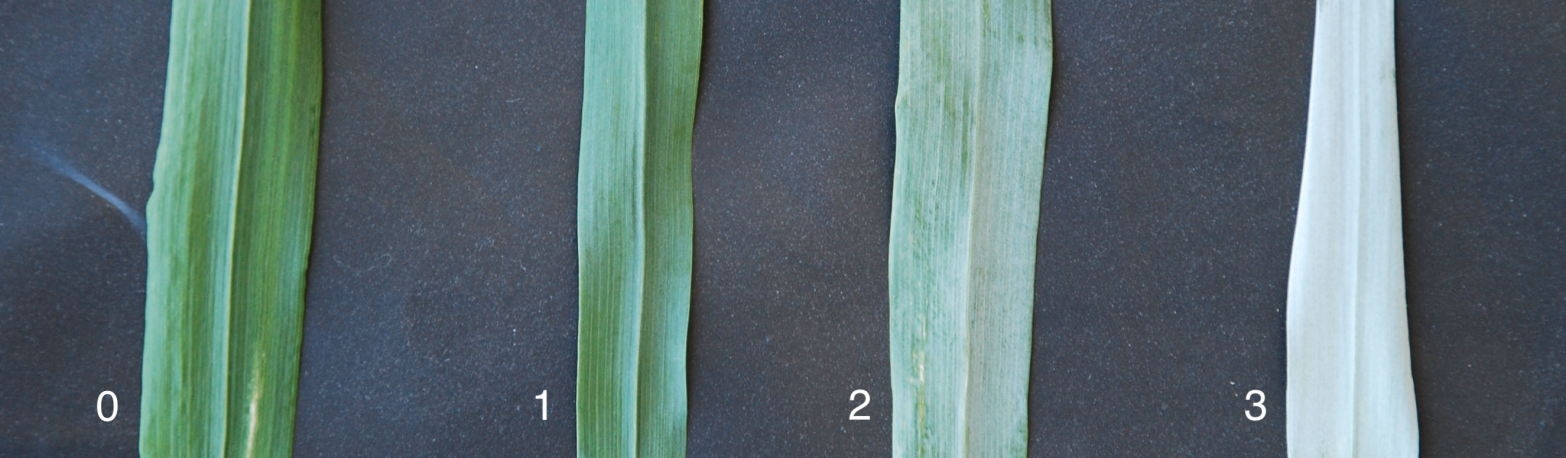
Glaucousness scale, where 0 is non-glaucous, 1 is a bit glaucous, 2 is quite glaucous, and 3 is abundant glaucousness.

Glaucousness was evaluated by visually scoring different parts of the plant: stem (Gst), flag leaf abaxial (Gab) and adaxial (Gad) sides, and the spike (Gsp). The scale used was 0 to 3 (none to abundant glaucousness) as shown in **Figure 1**. The evaluation was performed in the main tiller of three randomly selected plants per plot.

#### 2.4.2 Leaf characterisation

Leaf width (LW) and length (LL) were measured in the flag leaves of three random tillers per plot using a digital Vernier scale. Leaf width was measured in the widest part of the leaf, and the length was measured along the blade axis. Leaf angle (LA) and rolling (LR) along the blade axis of the leaf were visually scored. Angles were scored in the flag leaves of three random tillers per plot at the heading stage (LAH; Zadoks stage 55) and flowering (LAF; Zadoks stage 65) in each plot. The measurements were performed by a scale depending on whether the angle was less than 45° or the leaf was totally vertical (0–6 rate). Leaf rolling was visually assessed at the plot level, estimating the average degree of rolling of flag leaves along their longitudinal axis. A 0–3 scale was used, where 0 = fully flat (no rolling), 1 = slight rolling, 2 = moderate rolling with both margins curved inward but not touching, and 3 = complete rolling with the margins touching or overlapping. The percentage of the plot exhibiting rolling (LRp) was also evaluated.

#### 2.4.3 Canopy height

Canopy height (CH) was measured using a ruler in the central part of the plot to avoid edges. A flat piece of 30 × 30 cm of 2-mm-thick cardboard was placed on top of the canopy, and the height was measured from the ground to the cardboard.

#### 2.4.4 Normalised difference vegetation index

To estimate the amount of green canopy cover, NDVI was calculated using a GreenSeeker Handheld Crop Sensor (NTech Industries, Ukiah, CA, Canada) on sunny, calm days around noon when most genotypes had reached heading (Zadoks stage 55), flowering (Zadoks stage 65), and physiological maturity (Zadoks stage 92) following the procedures described in Ullah et al. (2020).

#### 2.4.5 Light interception monitoring

In selected genotypes, light interception was recorded every 2 minutes during the day using PARbars described by Salter et al. (2020). The PARbars were installed horizontally on the ground under the canopy for a 24-hour period in the same three plots of the trial where albedometers had been installed. For analysis, the average of the data collected around noon (from 11 am to 1 pm) was used.

#### 2.4.6 Phenology

Days to heading (DH) was determined as the number of days from sowing to the date when 50% of the plot was at half spike emergence. Days to flowering (DF) was determined as the number of days from sowing to the date when 50% of the plants in the plot were flowering with visible anthers. Days to physiological maturity (DM) was determined as the number of days from sowing to the date when 50% of the plot had reached maturity. Maturity was estimated as the loss of green colour from the spike to the peduncle.

#### 2.4.7 Yield

All plots were reduced to 4-m length prior to harvest by removing 1 m at each end of each plot, resulting in a harvested area of 8 m^2^. All trials were harvested at maturity using a combine harvester. Every plot was automatically weighed, and the yield was calculated as t ha^−1^.

### 2.5 Air temperature in the canopy and canopy temperature

**Figure 2:**
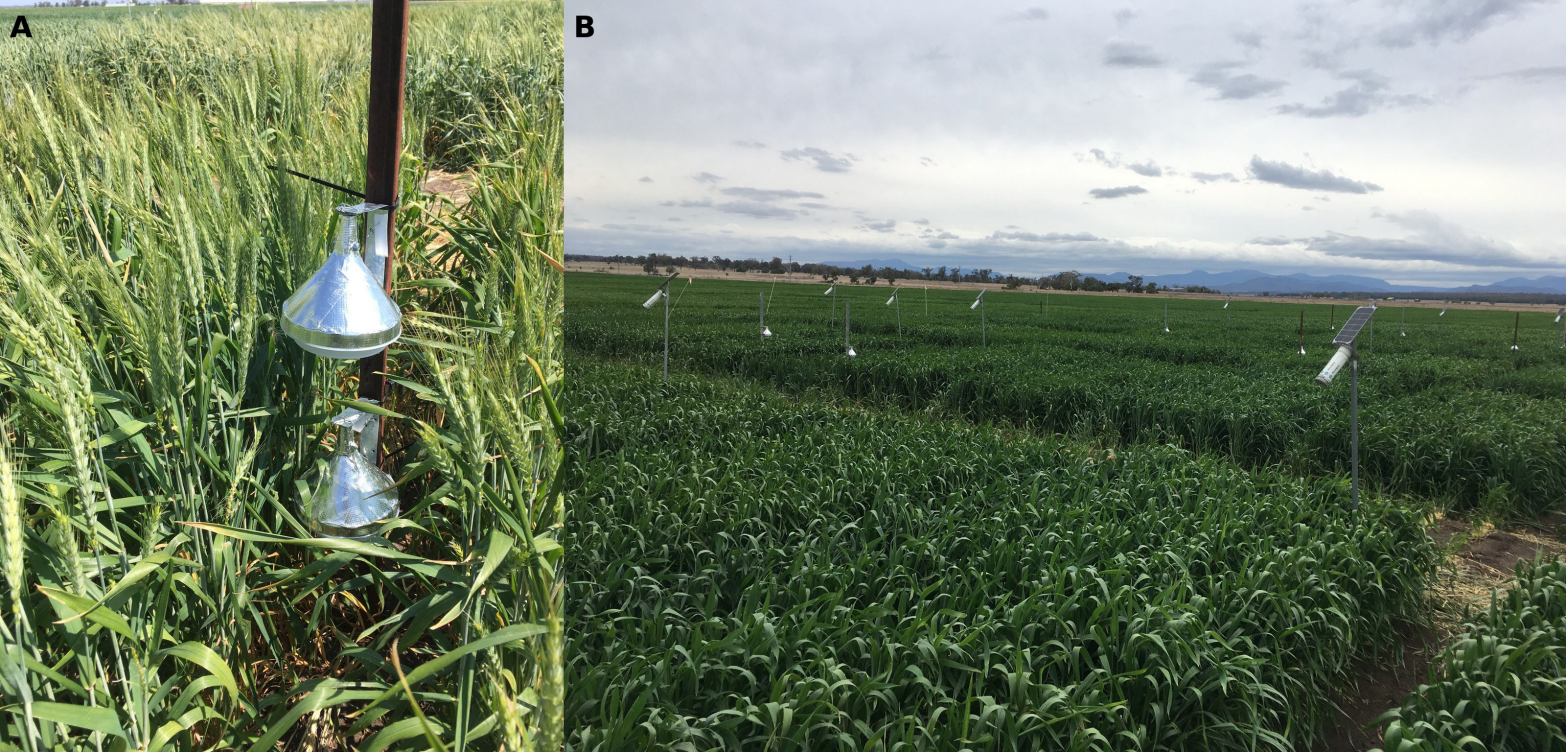
**(A)** iButtons and their sun shields installed at two heights in a wheat canopy. **(B)** Infrared sensors (Arducrops) installed in wheat plots for measurement of canopy temperature.

Air temperature was monitored in the canopy profile from spike emergence to maturity in six genotypes (including the three genotypes with albedometers) and three replicate plots. iButton^®^ DS1923 Hygrochron Temperature/Humidity Data Logger (Maxim Integrated, San José, CA, USA) devices were selected for this purpose because of their durability, self-sufficiency, small size (10-mm diameter), and low cost. They were installed at two different heights in the canopy: one at spike height and the other below the flag leaf. The loggers were protected by custom-built sun shields that provided enough air flow for ventilation and accurate temperature readings. Sun shields were designed based on Tarara and Hoheisel (2007) by adapting easily available low-cost materials (**Figure 2A**). Two plastic kitchen funnels were stacked, leaving a 5-mm gap to ensure air circulation. The upper funnel exposed to sun radiation was covered with aluminium sticky tape to reflect solar radiation, and the inside funnel was drilled several times to allow air movement around the logger hanging inside. This funnel combo was mounted on a plate forming a 90° angle so that it could be easily fixed to a post using zip ties. The loggers were installed in the field, and data were downloaded every 3 weeks using the 1-Wire^®^ Viewer open-source Software connected by a USB reader Blue Dot™ receptor (Maxim Integrated, San José, CA, USA).

Canopy temperature was monitored from spike emergence (Zadoks stage 55) to maturity (Zadoks stage 92) using infrared sensors (Arducrops^®^, CSIRO Plant Phenomics Centre, Canberra, Australia) provided and supported by the High Resolution Plant Phenomics Centre in Canberra across the 2017–2019 seasons. A data point was recorded every 15 minutes and automatically sent to a CSIRO data interface PhenoSmart^®^ (CSIRO Plant Phenomics Centre, Canberra, Australia). These sensors were fixed on a pole (**Figure 2B**) 60 cm from the top of the canopy, facing the plot from a corner at 45° to avoid soil exposure as much as possible, especially in open canopies. Since the infrared sensor’s angular field of view is 10°, the temperature recorded corresponds to the average temperature of the canopy included in the vision area.

### 2.6 Statistical analysis

Data management and statistical analyses were performed using Python (van Rossum and Drake, 2009) with the software libraries Pandas (McKinney, 2010), Seaborn (Waskom et al., 2017), Matplotlib (Hunter, 2007), and SciPy and Statsmodels (Seabold and Perktold, 2010). Fixed effects, random effects, and interactions were determined using linear mixed models with the package statgenSTA (van Rossum, 2024b) to fit spatial single trials, considering the experiment as a randomised complete block design using ASReml as the engine model and statgenGxE (van Rossum, 2024a) to fit genotype by environment interactions. Year, time of sowing, and genotype were set as fixed effects for multi-environment and single-environment analyses conducted as required (time of sowing within year). Replicates, rows, and ranges within times of sowing and years were considered random effects. Heritability was calculated using statgenSTA, setting genotype as a random effect for every environment (every year and time of sowing). The Wald statistics was computed from a model fitter for albedo (morning, noon, and afternoon) with fixed effects specified as Year * Environment + Genotype + Genotype: Environment, fitted using ASReml. To visualise genotype (G), environment (E), and their interaction (G × E), GGE biplots were constructed. These biplots were used to present the relationships between genotypes and environments (Yan et al., 2000).

## 3 Results

### 3.1 Albedometer measurements

**Figure 3:**
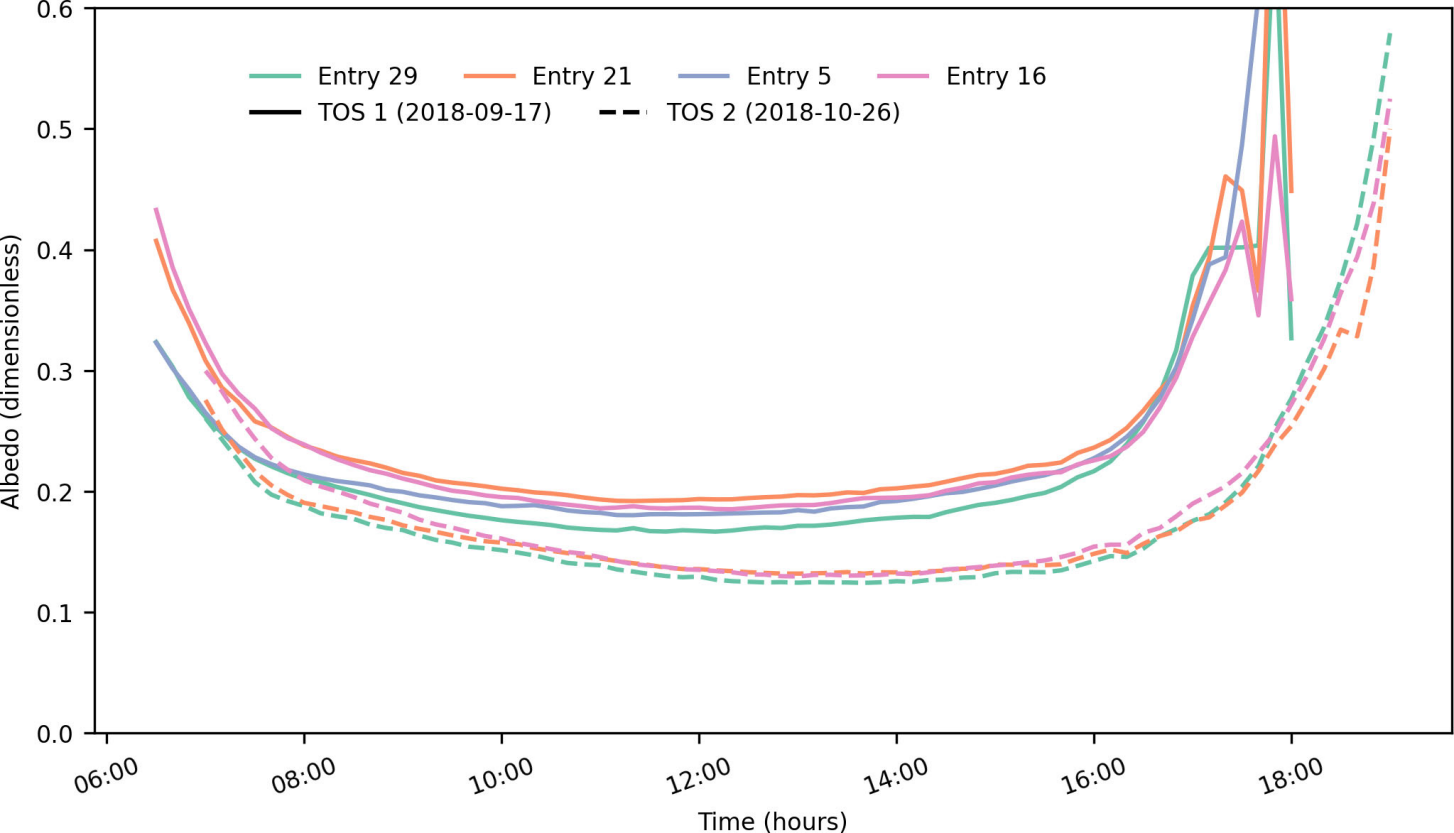
Canopy albedo over the course of a day at the flowering stage during the optimal time of sowing (TOS 1) and late time of sowing (TOS 2) for the four genotypes assessed (entries 29, 21, 5, and 16). Data shown is an example of representative data from individual plots of one replicate for each genotype.

Continuous measurements taken using albedometers showed variation during the day for both times of sowing (**Figure 3**). Minimum albedo values were reached around noon, and different genotypes had slightly different values consistently throughout the day. Albedo values were larger in TOS 1 than in TOS 2.

### 3.2 Estimation of canopy albedo from spectral reflectance

**Figure 4:**
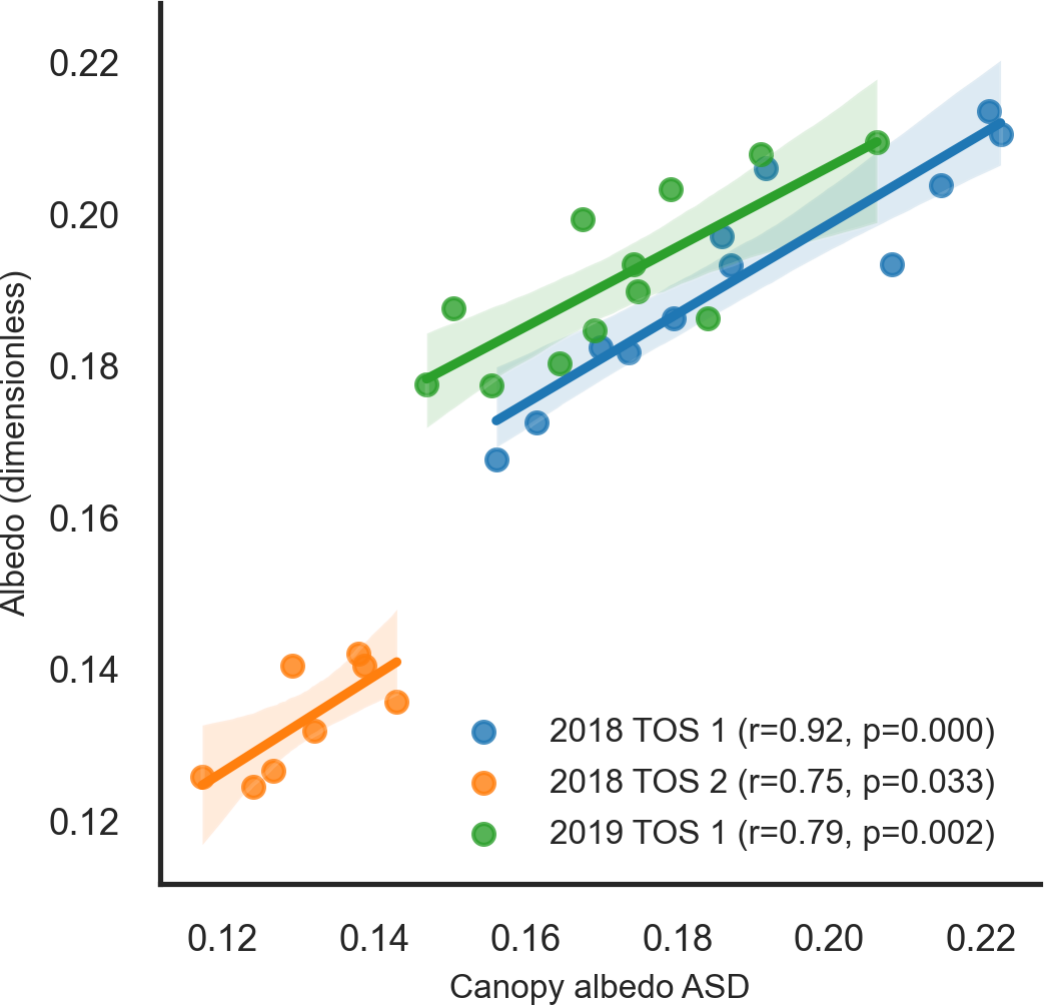
Correlations of canopy albedo at noon measured using spectroradiometer ASD and albedometer across three environments: time of sowing (TOS) 1 in 2018 and 2019 and time of sowing 2 in 2018 in Narrabri field trials (NSW, Australia). ASD, Analytical Spectral Devices.

Albedo from the albedometers and ASD were time-aligned. Albedo measured using the albedometer was consistently higher than that obtained using ASD, but values recorded by the two devices were more similar at noon (lower values in the graph or valleys; **Supplementary Figure S1** in **Supplementary Material**). Regression analysis found a significant correlation between albedometer and ASD data at noon (**Figure 4**). All three environments showed significant r values, indicating a strong correlation.

### 3.3 Canopy albedo heritability in different environments

The Wald statistics from multi-environment analysis of canopy albedo assessed for all 30 genotypes with the ASD and heritability values are shown in **Table 1**. Genotype main effects were significant for albedo for both TOS 1 and TOS 2 for all times of day, except for the afternoon of TOS 2 in 2018. Morning albedo was less significant (higher p-value) than at noon and in the afternoon. Heritability for albedo was generally lower in TOS 2 than in TOS 1 and ranged from 0.22 to 0.86. The highest heritability values for albedo were at noon in all environments.

**Table 1:** Wald statistics from multi-environment analysis of albedo across 2 years (2018 and 2019) and two times of sowing [optimum and late (TOS)] at three different times of the day and heritability values.

Year	TOS	Time	Wald	p-Value	Significance	Heritability
2018	1	Morning	2.579	0.0010712	**	0.61
2018	1	Noon	7.290	0.0000000	***	0.86
2018	1	Afternoon	3.722	0.0000102	***	0.73
2018	2	Morning	2.139	0.0069403	**	0.53
2018	2	Noon	3.472	0.0000272	***	0.71
2018	2	Afternoon	1.278	0.2109097	NS	0.22
2019	1	Morning	2.495	0.0015285	**	0.60
2019	1	Noon	6.831	0.0000000	***	0.85
2019	1	Afternoon	3.732	0.0000731	***	0.73

Significance values: *p < 0.05, **p < 0.01, ***p < 0.001, and NS, non-significant.

**Figure 5:**
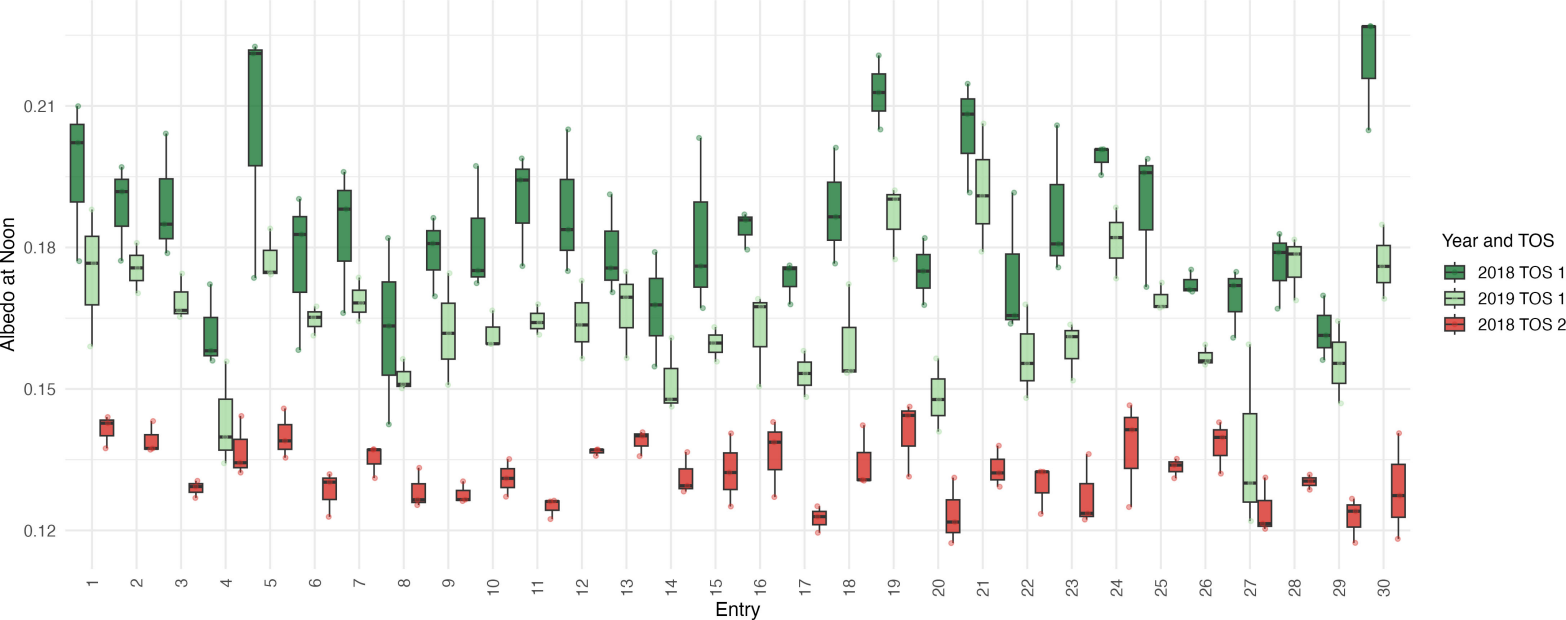
Canopy albedo at noon by genotype (entry number) measured using a spectroradiometer [Analytical Spectral Devices (ASD)] for optimal (TOS 1) and late time of sowing (TOS 2) in 2018 and optimal sowing in 2019. Data are means ± SEM of three replicate plots.

**Figure 5** shows the mean canopy albedo measured with ASD at noon by genotype, TOS, and year. Optimal sowing (TOS 1) had higher albedo values than late sowing (TOS 2) in 2018, whereas TOS 1 in 2019 was generally lower than TOS 1 in 2018. Genotypes had similar behaviour in all environments: entries 5, 19, 21, and 30 were generally the highest, and entries 4, 20, and 27 were the lowest across all TOS.

### 3.4 Multi-environment analysis of canopy albedo

**Figure 6:**
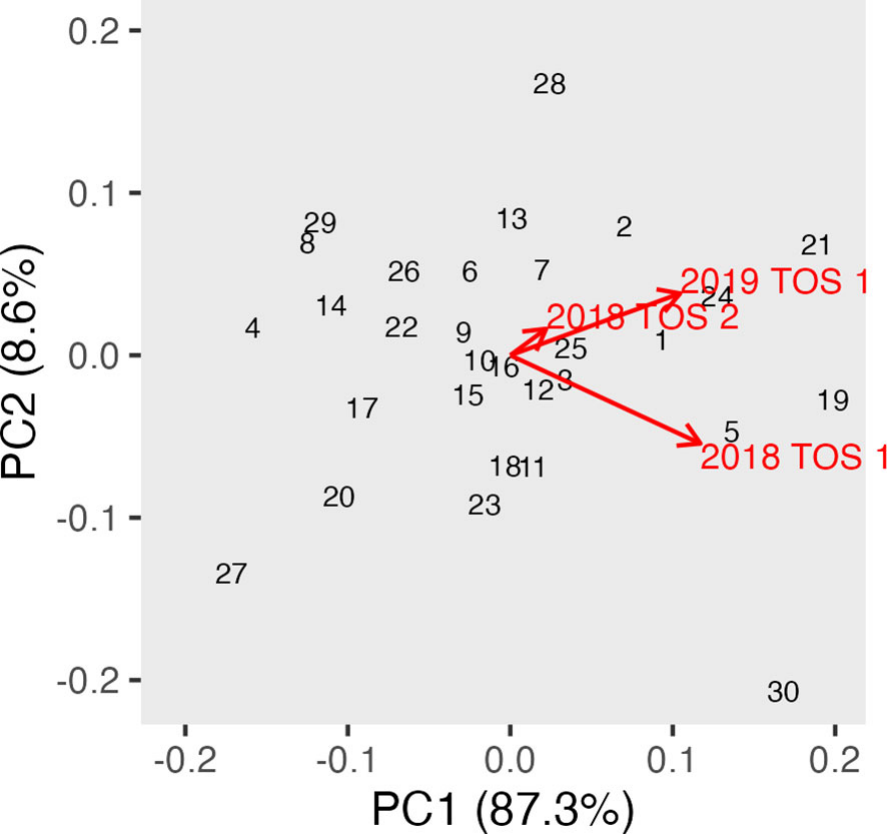
GGE biplot for albedo in the morning (measured using hyperspectral ASD on 30 genotypes in three replicate plots) and across three environments (TOS 1 and 2 in 2018 and TOS 2 in 2019). ASD, Analytical Spectral Devices.

**Figure 7:**
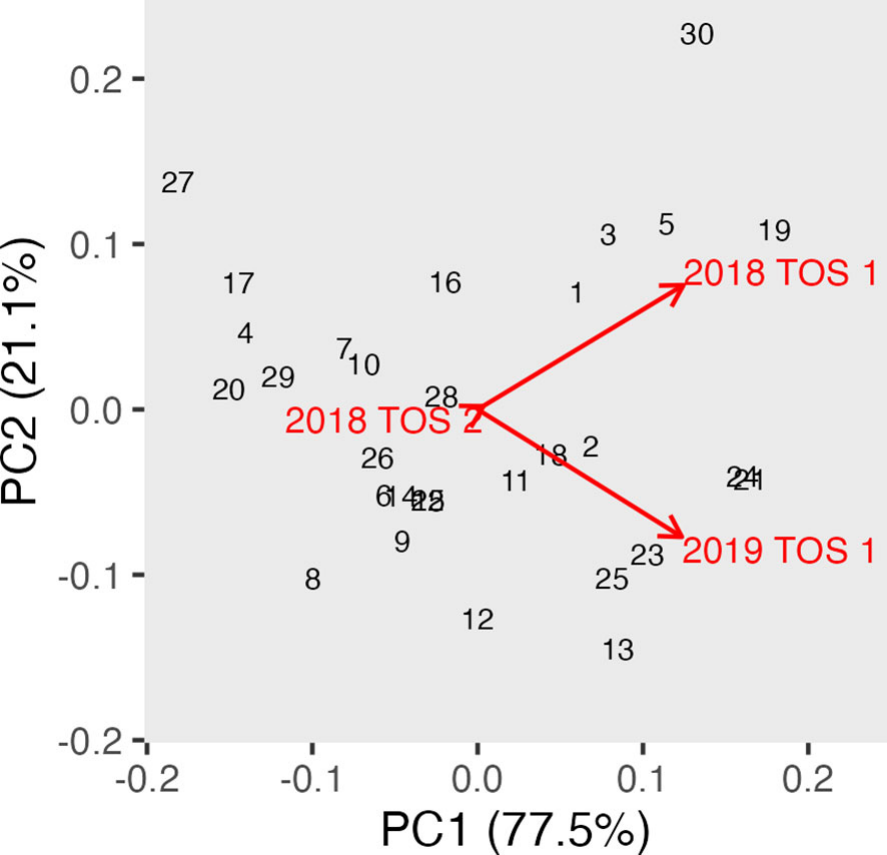
GGE biplot for albedo at noon (measured using hyperspectral ASD on 30 genotypes in three replicate plots) and across three environments (TOS 1 and 2 in 2018 and TOS 2 in 2019). ASD, Analytical Spectral Devices.

**Figure 8:**
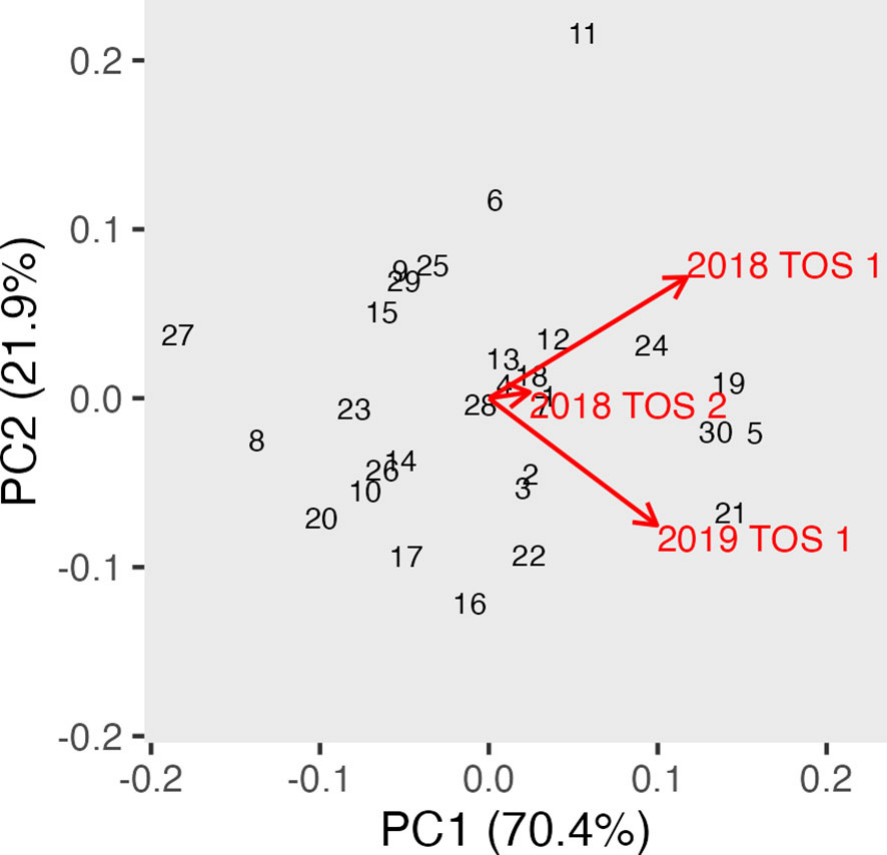
GGE biplot for albedo in the afternoon (measured using hyperspectral ASD on 30 genotypes in three replicate plots) and across three environments (TOS 1 and 2 in 2018 and TOS 2 in 2019). ASD, Analytical Spectral Devices.

Canopy albedo assessed using a spectroradiometer (ASD) was analysed using REML in a multi-environment approach to calculate albedo best linear unbiased prediction (BLUP). **Table 2** shows the Wald statistics for the fixed effects of year, trial, and genotype, as well as the interaction Genotype: Environment, across three times of day: morning, noon, and afternoon. The Wald test showed generally a highly significant effect of environment and year at all three times of the day. The genotype effect was also highly significant in all scenarios along the day. GGE biplots were constructed based on **Table 2** to visualise albedo in the morning, noon, and afternoon across different years and TOS (**Figures 6**–**8**). The three environments were all less than 90°, indicating a degree of relationship among all three variables. However, the TOS 2–2018 vector was the shortest, while TOS 1 (2018 and 2019) had longer vectors and therefore explained more of the G × E. Environments at noon tended to be more closely related.

**Table 2:** Wald statistics for albedo (ASD) from multi-environment analysis of albedo across 2 years (2018 and 2019) and two times of sowing [optimum and late (TOS)] at three different times of the day: morning, noon, and afternoon.

Time of day	Wald statistics			
	Year	Environment	Genotype	Genotype: Environment
Albedo morning	3.8*	548.4***	85.8***	61.9
Albedo noon	25.3***	1,310.4***	260.2***	112.4***
Albedo afternoon	42.5***	148***	90.3***	74.1

ASD, Analytical Spectral Devices.

Significance values: *p < 0.05, ***p < 0.001, and NS, non-significant.

### 3.5 Correlations among traits, temperature parameters, and albedo ASD

Correlations among different canopy and leaf traits and albedo measurements assessed using the ASD spectroradiometer were investigated using TOS in 2018 and 2019. A matrix of Pearson’s coefficients and their significance is given in **Supplementary Table S2** (**Supplementary Material**).

**Figure 9:**
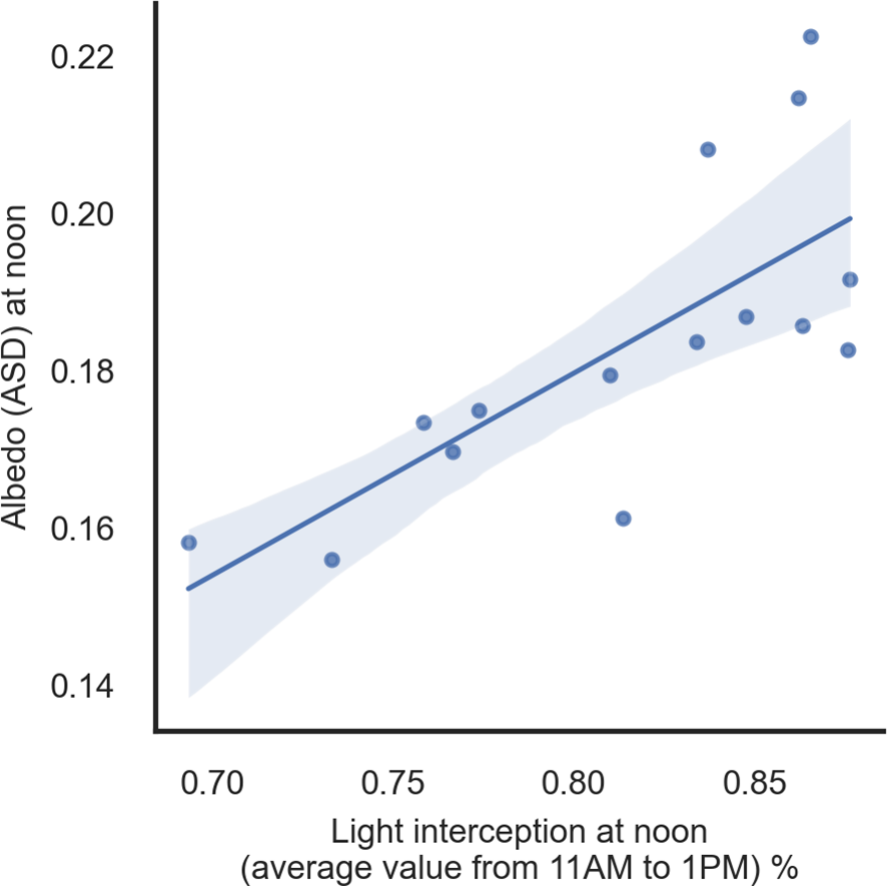
Linear regression of albedo (ASD) and light interception at noon (average value from 11 am to 1 pm) in 2018 for TOS 1 (r = 0.74, p < 0.01). ASD, Analytical Spectral Devices.

Some traits were significantly positively correlated with albedo ASD at noon, such as LL (0.68) and NDVI at flowering and maturity (approximately 0.5). Glaucousness on the abaxial side of the leaf tended to be correlated with albedo ASD, but the relationship varied between positive and negative associations depending on TOS, and r values were low. Phenology was also significantly correlated with albedo ASD, with the strongest associations when albedo was measured at noon. Light interception was significantly correlated with albedo ASD at noon in TOS 1 in 2018 (**Figure 9**).

**Figure 10:**
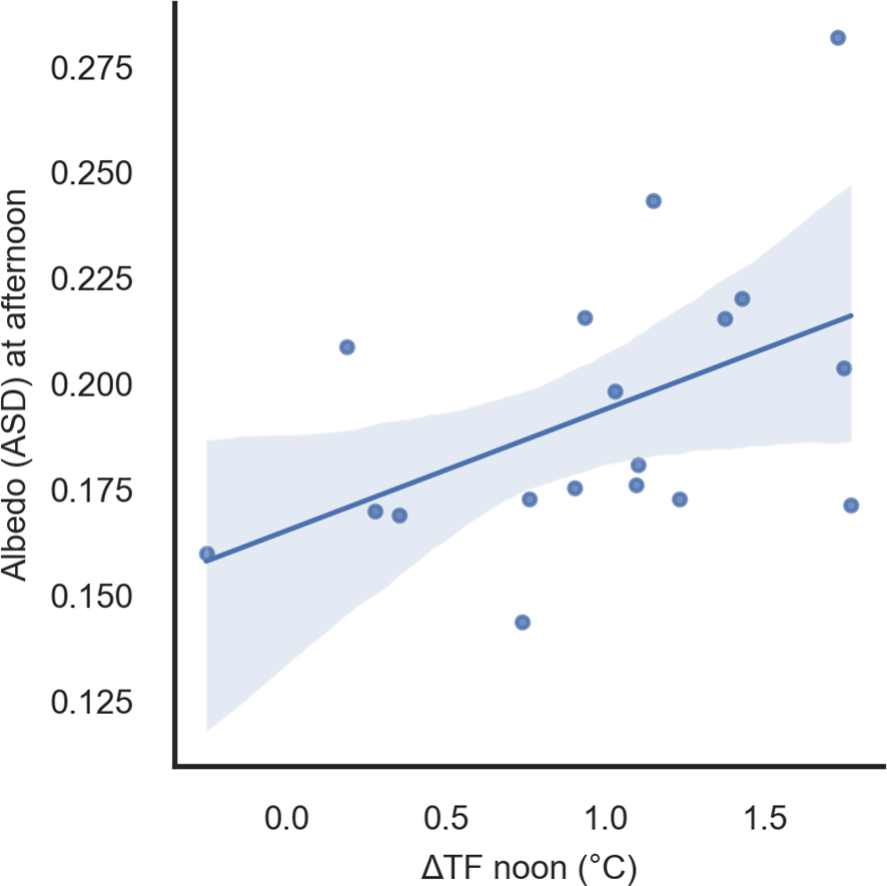
Linear regression of albedo (ASD) in the afternoon and ΔTF (air temperature gradient between leaf and spike at flowering) in the afternoon in 2018 for TOS 1 (r = 0.48, p < 0.01). ASD, Analytical Spectral Devices.

The gradient temperature between the flag leaf and the spike (ΔT) was generally correlated with albedo ASD (**Supplementary Table S2**). Stronger relationships were found at central times of the day, for example, between albedo in the afternoon and ΔTF (air temperature gradient at flowering) at noon in TOS 1 in 2018 (**Figure 10**; r = 0.48, p < 0.05) or albedo at noon and ΔTH (air temperature gradient at heading) plus ΔTF (air temperature gradient at flowering) in the afternoon in TOS 2 in 2018 (r = 0.56, p < 0.05; r = 0.54, p < 0.05).

**Figure 11:**
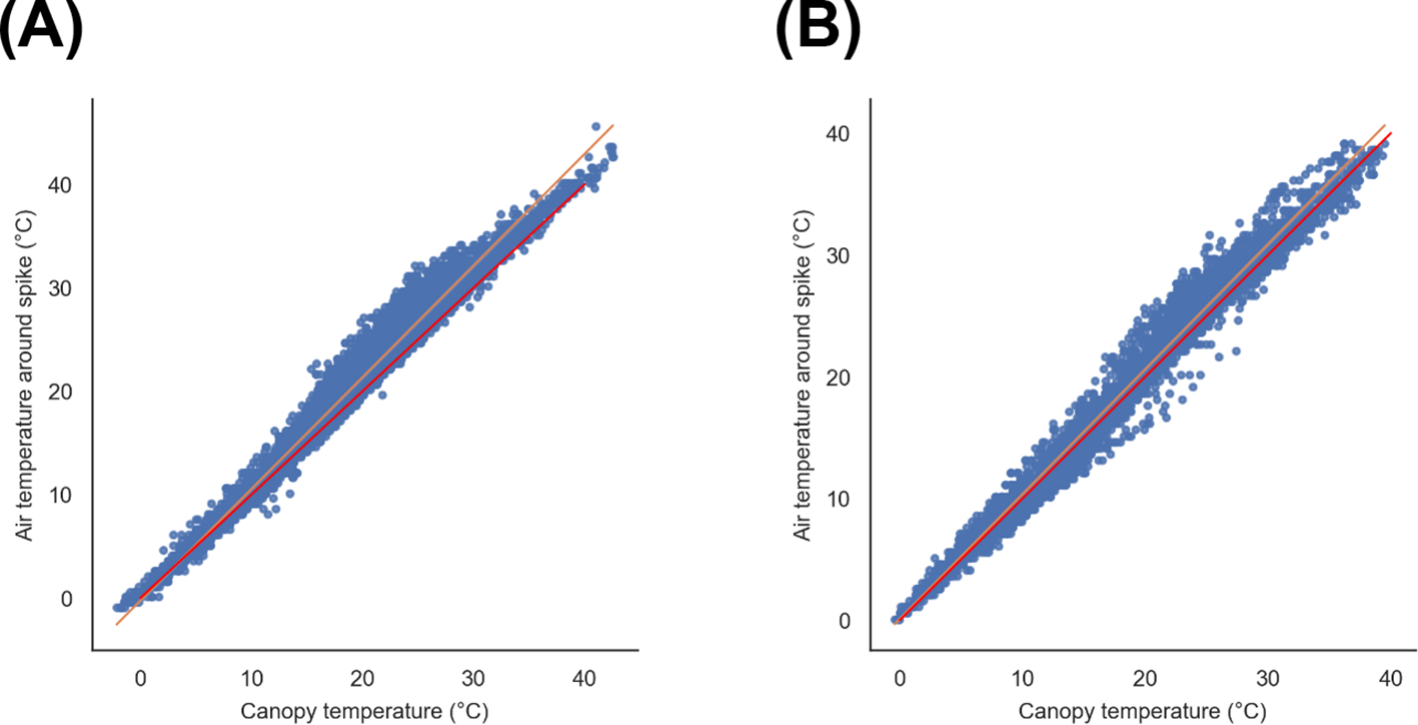
Examples of regression lines for canopy temperature and air temperature around the spike measured simultaneously in a plot across the season (from heading to maturity) in **(A)** 2018 and **(B)** 2019.

There were no significant correlations with canopy temperature and albedo (ASD) (data not shown). However, there was a strong relationship between canopy temperature (Arducrops) and air temperature around the spike (top iButton). These measurements were compared across the season, plot by plot, where both sensors were installed and recorded data simultaneously. Regression line parameters were described for each plot in **Supplementary Table S3**. These were all significant, and r values were very high. Two examples of the regression lines are shown in **Figure 11**.

## 4 Discussion

### 4.1 Development of a high-throughput method to measure albedo in field experiments

Albedo in crop canopies changes throughout the day depending on the interaction of the canopy with sun radiation. Therefore, albedo depends on sun position and canopy architecture, which in turn depends on several factors, including leaf distribution and leaf characteristics (Nagai, 2002). Potential links between albedo and heat tolerance or avoidance have been suggested (Lunagaria, 2021). In our study, we observed genotypic variation in both leaf and canopy traits related to heat tolerance (Yanli et al., 2007; Richards et al., 2014, 2019; Kiranakumara et al., 2024; Ponce de León and Bailey, 2024) and found that some of these traits were associated with differences in albedo. These results highlight the potential of albedo as an integrative trait for high-throughput phenotyping and genotypic selection under high-temperature conditions, especially as a heat-avoidance mechanism. While albedo can help reduce radiation load and potential overheating in the canopy (i.e., help tissue temperatures stay lower than air temperature), it is distinct from intrinsic heat-tolerance traits, which are physiological and biochemical responses that enable the plant to function under high temperatures (Farooq et al., 2011; Cossani and Reynolds, 2012). Albedo is influenced by differences in incident radiation and solar geometry as well as atmospheric conditions and canopy structure-related traits (Dexter et al., 2004; Nagai, 2002; Zhang et al., 2013; Drewry et al., 2014). Therefore, in breeding applications, albedo expression is context-dependent and should be used as a complement for other screening criteria, particularly in multi-environment trials where genotypes are evaluated across seasons and locations.

Albedo is usually time-consuming and costly to measure. Albedometers or pyranometers are expensive and need some expertise to handle and process the data. Moreover, they are stationary and designed to measure over long periods, and this makes them impractical for assessing large breeding experiments. In contrast, although spectroradiometers are also costly and require skill to use, they can quickly assess much larger numbers of genotypes: an advantage for breeding programs. This research focused on albedo at flowering, as this is the most critical period for heat stress damage, influencing yield (Farooq et al., 2011). Both methods of measuring albedo were used to design and validate a high-throughput method. Two main aspects were considered: i) the relationship between ASD measurements and those taken using albedometers to determine the reliability of ASD measurements and ii) the best time of the day to assess albedo for one-off measurements with an ASD in large experiments involving many genotypes. Results showed that noon was the most relevant time to measure albedo based on the consistent and stronger relationship between ASD and albedometer time series. At noon, solar radiation hits the canopy close to the zenith. Albedo asymmetries along the day due to differences between morning and afternoon solar angles, canopy water (e.g., dew), and crop developmental stage have been described by other authors (Dexter et al., 2004; Zhang et al., 2013). Lunagaria (2021) calculated albedo considering zenith angles; however, this was not considered here since the ASD pistol and albedometers were always facing perpendicular to the canopy surface and, therefore, more closely aligned with sun radiation at noon. This could explain why the ASD/albedometer relationship is more robust at noon than in the morning and afternoon. Our results showed that a high-throughput ground-based ASD method can be used to assess canopy albedo at flowering on large numbers of genotypes. The increased accuracy at noon is a possible advantage for the selection of heat-resistant genotypes, as heat stress is often most damaging when solar radiation is the highest (Zhang et al., 2022b). However, hyperspectral portable equipment is still expensive and requires regular calibration. Applications at large field-scale are limited by battery endurance and instrument weight (ergonomic issues for the operator). In addition, reference panel targets such as Spectralon^®^, used to quantify irradiance (solar radiation) and considered as near-Lambertian (homogeneous diffuse irradiance), need regular maintenance and calibration for accurate results (Anderson et al., 2002; Porcar-Castell et al., 2015). Therefore, although this screening method is effective, it has limitations. Scalability is needed for the method to be integrated into breeding pipelines where genotypic comparison of albedo in larger populations would be required. Solutions include incorporating the instrumentation on mobile platforms (Jimenez-Berni et al., 2018) and/or Unmanned Aerial Vehicle (UAV)-based reflectance sensing. Both of these technologies could be used to measure albedo as well as other relevant canopy and thermal traits at key developmental stages in practical breeding pipelines.

### 4.2 Canopy albedo: impact of time of sowing, genotypic variation, and albedo evolution

Because canopy albedo is a potential trait that can be used to differentiate genotypes in heat stress experiments, a multi-environment analysis was performed to evaluate the impact of TOS on canopy albedo (measured three times daily using a full-range spectroradiometer). The Multi Environment Trials (MET) results showed that TOS and genotype effects were significant. Albedo was higher in early TOS and related to fuller canopies and longer vegetative periods pre-flowering. In TOS 2, where the crops experienced higher temperatures from late sowing and thus, a shortened season, canopies were shorter and more open. Thus, the albedo range in TOS 1 (2018 and 2019) was larger than that in TOS 2 (2019) since genotypes were under increased heat stress at flowering (ranging from 22 °C to 24 °C in optimal sowing against 26 °C to 28 °C in late sowing). Light intercepted by the canopy, assessed simultaneously using PARbars, was lower for all genotypes at late sowing, indicating that the canopy was less dense and allowed greater light penetration. The smaller canopy in late sowing is typical of non-optimal growing seasons in northwestern New South Wales (Jugal K. Mani et al., 2013). Openness and size of the canopy play an important role in the penultimate leaf photosynthetic capacity and yield determination (Pearcy et al., 1989; Salter et al., 2020).

Albedo evolution throughout the day conformed to a U-shaped curve, where the minimum was around midday and increased dramatically at the beginning and end of the day, driven by changes in solar angle and, likely, canopy dynamics (e.g., dew presence, leaf angle adjustments, and/or the degree of leaf rolling). When analysing environments independently, the most significant genotypic differences in albedo were obtained for noon. The albedo of genotype entries 19 (commercial cultivar EGA Gregory), 21 (commercial cultivar Sunlin), and 5 (a breeding line) was consistently higher than that of other genotypes. Among these, entry 21 and entry 19 are commercial varieties adapted to the northwestern New South Wales growing region and are characterised by floppy flag leaves, whereas genotype 5 is a derivative of PBW343, a heat-resistant variety from India, characterised by thin, long flag leaves. Clearly, genotypic differences for canopy albedo were significant in the materials evaluated, and this was even more noticeable when solar radiation was intercepting the canopy closer to perpendicular. This could be due to solar angle and therefore radiation–equipment interaction since the equipment was installed in a fixed position but also linked to canopy changes under more severe conditions (extremely warm and dry environments such as Narrabri). Similarly, Nagai (2002) demonstrated that considering the directional distribution of radiation, rather than assuming isotropic conditions, improves the representation of canopy–atmosphere interactions. Differences among genotypes were significant in all scenarios, except for the afternoon measurements in TOS 2 (2018), probably due to windy conditions and high clouds that were not present in the morning and noon, so radiation was sub-optimal, and leaf movements likely distorted the results. Heritability for albedo was the highest for TOS 1 in 2018 (0.86) and 2019 (0.85); thus, genotypic effects tended to be more important than the environment in this study. This probably explains why the albedo range was larger in early sowing when temperatures during the flowering stage of development were closer to the optimum, even though genotypic differences remained significant in late sowing. Consequently, based on this set of wheat genotypes, phenotyping for albedo as a heat avoidance trait should be assessed at noon in optimally sown experiments.

### 4.3 Correlation of albedo with light interception, leaf characteristics, and temperature parameters

Some field traits, such as LL, canopy cover (NDVI), and phenology, correlated with albedo measurements (ASD) at noon. Phenology varied among genotypes and was also correlated with albedo at noon. Frumau and Vugts (1996) found a similar relationship between longer vegetative periods and higher albedo at flowering. LL, NDVI, and DF were significantly correlated, indicating that genotypes with longer vegetative periods develop more biomass. This correlation was not as strongly expressed in late sowing, where genotypic differences were less. The relationships between albedo, LL, and NDVI can be explained by Richards et al. (2019), who found that planophile canopies tend to have more floppy and longer leaves, compared with leaves in erectophile canopies. Hence, the canopies with higher albedo in this study possibly had more planophile canopy profiles.

Other leaf parameters, such as glaucousness, leaf width, leaf angle, and canopy height, did not show a strong relationship with albedo in this study. Although some of them showed statistically significant correlations, the corresponding r values were low, unlike previous studies that found that leaf angle, leaf rolling, and glaucousness can affect reflectance or related spectral indices in wheat (Richards et al., 1986; Wenjiang et al., 2008; Thippeswamy et al., 2022). Under our population and measurement conditions, however, these traits had limited influence on overall canopy reflectance. This difference may reflect the broader genetic diversity and the field environment used in our study, where canopy albedo integrates multiple structural and morphological components. In contrast, the mentioned studies often examined fewer genotypes or were performed under controlled conditions, where individual leaf traits have a stronger and more isolated effect on reflectance. Consequently, the expression and utility of albedo as a heat−avoidance trait will vary across environments and measurement conditions.

Light absorption patterns throughout the day were related to albedo, reaching a minimum around noon (full daily data not shown). Light interception has been related to canopy structure previously (Cossani and Reynolds, 2012; Ponce de León and Bailey, 2024). However, it needs to be remembered that the sensors were installed in the direction of the sun zenith and did not change their position throughout the day. Albedometers that follow the sun’s direction have been reported as desirable for more accurate data capture (Pinter et al., 1985; Lunagaria, 2021), but these are not feasible for phenotyping large numbers of genotypes. Interestingly, albedo assessed at noon, when sensors are aligned with sun radiation angle, was significantly correlated with light interception (r = 0.74). Salter et al. (2020) found a genotype influence on the light profile in the canopy, relative to photosynthesis using PARbars to quantify light interception. We also found significant genotype effects on light interception when noon hours only were considered. Therefore, the correlation between albedo and light interception supports the idea that albedo is linked to canopy structure and therefore heat avoidance, making it a potentially useful trait for breeding heat-tolerant wheat. Overall, canopy structure is likely to be the main driver of canopy albedo rather than leaf optical or morphological properties.

There is no clear evidence of a relationship between canopy temperature and albedo in this study. The use of dual sensors, as is the case for iButtons, seems to be a more effective way of normalising measurements at the plot level. Measuring air temperature at two levels also provided a better characterisation of the canopy architecture and its influence on the temperature profile, resulting in a better prediction tool for determining phenotypic differences. Therefore, albedo should be used as complementary information as a characterisation of the canopy microclimate driven by the incoming radiation.

The most relevant significant correlation found in this research was the air temperature difference between upper and lower iButton (ΔT) and albedo (from the ASD) at noon during flowering. This indicates that the ability of the canopy to reflect radiation during the most critical period for heat stress damage is related to larger differences in air temperature throughout the canopy profile. Since air temperature around the spike and canopy temperature were strongly correlated, ΔT offers a useful indicator for understanding how canopy architecture interacts with incoming radiation. Hence, considering albedo together with CT or ΔT provides a more complete description of the canopy microclimate.

Albedo was found to be a genetically diverse trait and related to components of canopy architecture. However, no clear relationship between CT and albedo was detected for this population under our measurement conditions, especially since CT and ΔT were assessed only in a subset of genotypes to allow continuous measurements. A drone equipped with thermal cameras can be used in combination with the albedo phenotyping approach described in this study. In this framework, albedo characterises the radiation−driven component of the canopy microclimate, whereas CT and CTD primarily reflect evaporative and physiological cooling linked to transpiration and plant water status. More research using a larger population is needed to better assess the link between albedo, CT, and potential implications for improving genotype performance in warming environments (Ridgwell et al., 2009).

## Data Availability

The raw data supporting the conclusions of this article will be made available by the authors, without undue reservation.
