# Supplementary material for: A wheat canopy albedo high-throughput phenotyping method and its relationship with canopy architecture and leaf properties
Source: Front Plant Sci. 2026 Feb 25;17:1769886. doi: 10.3389/fpls.2026.1769886 (PMC12975993; doi:10.3389/fpls.2026.1769886)
Supplement: Supplementary file 1 [file DataSheet1.docx]

Supplementary Material

## Supplementary Figures


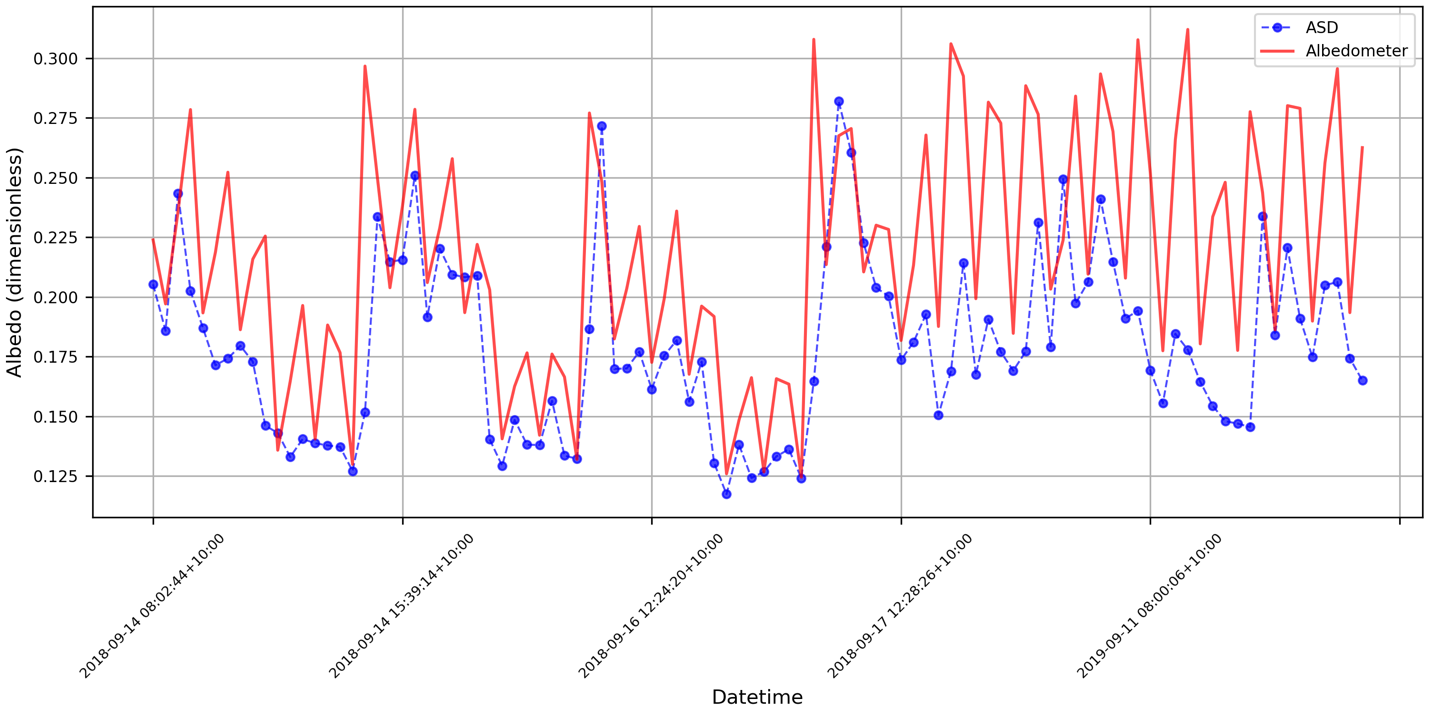


**Supplementary Figure 1.** Example relationship for visualisation purposes of canopy albedo from spectroradiometer (ASD) (morning, noon and afternoon) and corresponding albedometer measurements across all repetitions, years and times of sowing.

## Supplementary Tables

**Supplementary Table 1**. Genotypes description

| Genotype /Entry number | Variety name / Designation | Pedigree |
| --- | --- | --- |
| 1 | PBI09C004-BC-DH43 | BERKUT/2/BERKUT / 35883 M500110 |
| 2 | PBI09C009-BC-DH51 | SOKOLL/2/SOKOLL / 35888 M 500132 |
| 3 | PBI09C026-BC-DH41 | WAXWING*2/KIRITATI /3/WAXWING*2/KIRITATI /2/ 35888 M 500132 |
| 4 | ACIAR09PBI C04-17C-DH10 | PBW550//C80.1/*2Batavia |
| 5 | ACIAR09PBI C38-115C-DH9 | PBW343+L24+LR28/LANG |
| 6 | ACIAR09PBI C29-51C-DH1 | DBW16/Sunstate |
| 7 | ACIAR09PBI C27-0C-0N-3N | DBW16/ANNUELLO |
| 8 | ACIAR09PBI C26-0C-0N-2N | DBW16/GLADIUS |
| 9 | PBI07C101-DH64 | ISR 812.8/CARINYA |
| 10 | PBI07C101-DH154 | ISR 812.8/CARINYA |
| 11 | PBI07C201-BC-DH66 | VENTURA/IDO 637//VENTURA |
| 12 | CMSA06M00431S-040ZTM-040ZTY-31ZTM-04Y-0B | D67.2/P66.270//AE.SQUARROSA (320)/3/CUNNINGHAM/4/VORB |
| 13 | CMSA06M00128T-029(PINBD1BHET)Y-040ZTM-029(PINBD1BPOS)ZTY-67ZTM-0Y-0B | SLVS/ATTILA//WBLL1*2/3/GONDO/CBRD |
| 14 | ICW02.00099-11APTS-0AP-0AP-9AP-0AP | HUBARA-8/3/MON'S'/ALD'S'//BOW'S' |
| 15 | CMSA08M00008T-040Y-050ZTM-050Y-13ZTM-010Y-0B | RAC 1192/4/2*ATTILA/3/WEAVER*2/TSC//WEAVER |
| 16 | PBI09C009-BC-DH56 | SOKOLL/2/SOKOLL / 35888 M 500132 |
| 17 | CORAK | CORAK |
| 18 | SUNTOP | SUNTOP |
| 19 | EGA GREGORY | EGA GREGORY |
| 20 | MACE | WYALKATCHEM/STYLET//WYALKATCHEM |
| 21 | SUNLIN | SUNLIN |
| 22 | CMSS05B00663S-099Y-099M-099Y-099ZTM-13WGY-0B | ATTILA/3*BCN//BAV92/3/TILHI/5/BAV92/3/PRL/SARA//TSI/VEE#5/4/CROC_1/AE.SQUARROSA (224)//2*OPATA |
| 23 | SUNMATE | SUNMATE |
| 24 | COOLAH | COOLAH |
| 25 | ICW02.00478-3APTS-0AP-0AP-18AP-0AP | SERI 82/SHUHA'S'//CM85295-0101TOPY-2M-0Y-0M-3Y-0M-0AP |
| 26 | CMSA05Y01186T-040M-040ZTP0Y-040ZTM-040SY-12ZTM-03Y-0B | SOKOLL*2/TROST |
| 27 | ACIAR09PBI C15-0C-0N-2N | DBW14/C80.1/2*SR2 BATAVIA |
| 28 | ACIAR09PBI C38-150C-DH9 | PBW343+L24+LR28/LANG |
| 29 | GLADIUS | GLADIUS |
| 30 | MAGENTA | MAGENTA |
| 31 | YITPI | YITPI |
| 32 | LRPB TROJAN | LRPB TROJAN |
| 33 | MITCH | MITCH |
| 34 | LANCER | LANCER |

**Supplementary Table 2**. Trait-albedo (⍺) Pearson correlation table (r values) for optimal time of sowing (TOS 1) and late time of sowing (TOS 2) in 2018 and TOS 1 in 2019 in Narrabri, NSW. GAD: glaucousness adaxial side of the leaf (0-3 rating); GAB: glaucousness abaxial side of the leaf (0-3 rating); GST: glaucousness stem (0-3 rating); GSP: glaucousness spike (0-3 rating); LW: leaf width (cm); LAH: leaf angle at heading (0-6 rating); LAF: leaf angle at flowering (0-6 rating); LRP: leaf rolling considering portion of plot full plot (0-3 rating); LR: leaf rolling (0-3 rating); LL: leaf length; CHF: canopy height at flowering (cm); CHH: canopy height at heading (cm); CHM: maximum canopy height (cm); NDVIF: Normalized Difference Vegetation Index at flowering; NDVIH: Normalized Difference Vegetation Index at heading; NDVIM: Normalized Difference Vegetation Index at maturity; DF: days to flowering; DH: days to heading; DM: days to maturity; ∆T: temperature difference between upper and lower iButtons; LI: light interception at flowering around noon measured using PARbars. Significance values: *: P<0.05, **: P<0.01, ***: P<0.001 and NS: non-significant

|  | ⍺ morning 2018 TOS 1 | ⍺ noon 2018 TOS 1 | ⍺ afternoon 2018 TOS 1 | ⍺ morning 2018 TOS 2 | ⍺ noon 2018 TOS 2 | ⍺ afternoon 2018 TOS 2 | ⍺ morning 2019 TOS 1 | ⍺ noon 2019 TOS 1 | ⍺ afternoon 2019 TOS 1 |
| --- | --- | --- | --- | --- | --- | --- | --- | --- | --- |
| Gad | 0.11 | -0.11 | -0.1 | 0.24** | -0.04 | -0.22** | 0 | 0.03 | 0.01 |
| Gab | -0.26** | -0.18* | -0.22** | -0.13 | -0.17 | 0.32*** | 0.22** | 0.2* | 0.18* |
| Gst | -0.15 | -0.08 | -0.08 | -0.1 | -0.29*** | 0.04 | 0.06 | -0.1 | -0.1 |
| Gsp | -0.02 | 0.11 | 0.13 | -0.03 | -0.18* | 0.04 | 0.11 | -0.02 | 0.02 |
| LW | 0 | 0.04 | 0.1 | -0.09 | -0.05 | 0.04 |  |  |  |
| LAH | 0.25** | 0.23** | 0.24** | 0.15 | 0.05 | 0.11 |  |  |  |
| LAF | -0.02 | -0.17 | -0.14 | 0.14 | 0.28*** | -0.09 |  |  |  |
| Yield | 0.18* | 0.27*** | 0.28*** | -0.23** | 0.09 | 0 | 0.06 | -0.04 | -0.12 |
| LRp | -0.25** | -0.28*** | -0.23** | 0.24** | -0.01 | -0.1 | -0.1 | -0.22** | -0.08 |
| LR | -0.23** | -0.32*** | -0.26** | 0.22** | -0.07 | -0.01 | -0.15 | -0.27** | -0.18* |
| LL | 0.4** | 0.68*** | 0.59*** | 0.1 | 0.55** | 0.1 |  |  |  |
| DH | 0.46*** | 0.66*** | 0.66*** | 0.15 | 0.38*** | -0.27** | 0.44*** | 0.72*** | 0.45*** |
| DF | 0.43*** | 0.65*** | 0.64*** | 0.17 | 0.39*** | -0.24** | 0.47*** | 0.75*** | 0.42*** |
| DM | 0.34*** | 0.61*** | 0.59*** | -0.02 | 0.23** | -0.23** | 0.38*** | 0.58*** | 0.27** |
| CHH | 0.01 | -0.04 | -0.05 | 0.23** | 0.15 | 0.15 | -0.18* | -0.27** | -0.01 |
| CHF | -0.08 | -0.14 | -0.13 | 0.31*** | 0.36*** | 0 | -0.24** | -0.29*** | -0.01 |
| CHM | 0.25** | 0.27*** | 0.31*** | 0.33*** | 0.44*** | 0 | 0.03 | 0.12 | 0.24** |
| NDVIH | 0.35*** | 0.29*** | 0.34*** | 0.06 | 0.34*** | -0.1 | 0.28*** | 0.43*** | 0.41*** |
| NDVIF | 0.42*** | 0.32*** | 0.38*** | 0.15 | 0.17 | -0.03 | 0.39*** | 0.57*** | 0.34*** |
| NDVIM | 0.47*** | 0.5*** | 0.51*** | 0.05 | 0.17 | -0.01 | 0.39*** | 0.61*** | 0.35*** |
| ∆TH noon | 0.01 | 0.25 | 0.31 | 0.12 | 0.51* | -0.19 | 0.28 | 0.02 | 0.02 |
| ∆TH midnight | 0.02 | -0.22 | 0.01 | 0.3 | 0.12 | -0.49 | -0.01 | -0.03 | -0.03 |
| ∆TH afternoon | 0 | 0.1 | 0.26 | 0.29 | 0.56* | -0.34 | 0.46* | 0.34 | 0.19 |
| ∆TF noon | 0.06 | 0.41* | 0.48** | 0.1 | 0.5* | -0.26 | 0.38 | 0.09 | 0.03 |
| ∆TF midnight | -0.01 | -0.21 | -0.09 | 0.28 | 0.08 | -0.44 | -0.01 | -0.06 | -0.07 |
| ∆TF afternoon | 0.03 | 0.31 | 0.44* | 0.27 | 0.54* | -0.34 | 0.45* | 0.32 | 0.15 |
| ∆TM noon | -0.09 | 0.45* | 0.32 | -0.21 | -0.01 | -0.19 | -0.15 | -0.35 | -0.25 |
| ∆TM midnight | 0.22 | 0.24 | 0.22 | 0.1 | -0.1 | -0.21 | 0.2 | 0.06 | 0.17 |
| ∆TM afternoon | 0.13 | 0.52** | 0.41* | 0.06 | -0.15 | -0.4 | -0.18 | -0.26 | -0.19 |
| LI |  | 0.74*** |  |  | 0.32 |  |  | -0.14 |  |

**Supplementary Table 3**. Regression line parameters from comparisons of canopy temperature data (Arducrops) and air temperature around the spike (top iButton) across the season by plot in 2018 and 2019.

| Plot ID^1^ | Year | Slope | Intercept | *r* | P value | Standard error |
| --- | --- | --- | --- | --- | --- | --- |
| 2018124003 | 2018 | 1.080 | -0.245 | 0.974 | 0.000 | 0.002 |
| 2018121003 | 2018 | 1.065 | -0.208 | 0.980 | 0.000 | 0.002 |
| 2018119003 | 2018 | 1.081 | 0.264 | 0.983 | 0.000 | 0.002 |
| 201816002 | 2018 | 1.085 | 0.516 | 0.974 | 0.000 | 0.002 |
| 2018121004 | 2018 | 1.054 | 0.212 | 0.975 | 0.000 | 0.002 |
| 2018121005 | 2018 | 0.993 | 1.486 | 0.967 | 0.000 | 0.002 |
| 2018120005 | 2018 | 1.037 | 0.314 | 0.979 | 0.000 | 0.002 |
| 201819005 | 2018 | 1.076 | 0.796 | 0.976 | 0.000 | 0.002 |
| 201817005 | 2018 | 1.095 | 0.659 | 0.977 | 0.000 | 0.002 |
| 201816005 | 2018 | 1.054 | 0.856 | 0.979 | 0.000 | 0.002 |
| 201815004 | 2018 | 1.060 | -0.187 | 0.977 | 0.000 | 0.002 |
| 201819004 | 2018 | 1.058 | 0.240 | 0.979 | 0.000 | 0.002 |
| 2018110004 | 2018 | 1.043 | 0.028 | 0.980 | 0.000 | 0.002 |
| 201818003 | 2018 | 1.069 | -0.346 | 0.976 | 0.000 | 0.002 |
| 201815002 | 2018 | 1.077 | 0.098 | 0.986 | 0.000 | 0.002 |
| 201817001 | 2018 | 1.074 | 0.665 | 0.979 | 0.000 | 0.002 |
| 201814001 | 2018 | 1.093 | 0.624 | 0.972 | 0.000 | 0.002 |
| 201811001 | 2018 | 1.056 | 0.282 | 0.979 | 0.000 | 0.002 |
| 2019318005 | 2019 | 1.026 | 0.099 | 0.976 | 0.000 | 0.003 |
| 2019319003 | 2019 | 1.029 | 0.327 | 0.967 | 0.000 | 0.003 |
| 2019321002 | 2019 | 1.048 | 1.117 | 0.981 | 0.000 | 0.002 |
| 2019321003 | 2019 | 1.030 | 1.694 | 0.972 | 0.000 | 0.003 |
| 2019321005 | 2019 | 1.017 | 1.764 | 0.969 | 0.000 | 0.003 |
| 2019322005 | 2019 | 1.015 | 1.352 | 0.968 | 0.000 | 0.003 |
| 2019324004 | 2019 | 1.017 | 1.488 | 0.978 | 0.000 | 0.002 |
| 201912004 | 2019 | 1.063 | 1.213 | 0.918 | 0.000 | 0.004 |
| 201914004 | 2019 | 1.158 | 1.823 | 0.174 | 0.000 | 0.216 |
| 201915003 | 2019 | 1.081 | 0.472 | 0.923 | 0.000 | 0.004 |
| 201916001 | 2019 | 1.060 | 0.441 | 0.920 | 0.000 | 0.004 |
| 201916003 | 2019 | 1.081 | 0.074 | 0.920 | 0.000 | 0.004 |
| 201917004 | 2019 | 1.072 | -0.107 | 0.921 | 0.000 | 0.004 |
| 201918004 | 2019 | 1.055 | 0.265 | 0.924 | 0.000 | 0.004 |
| 2019110002 | 2019 | 1.086 | 0.592 | 0.924 | 0.000 | 0.004 |
| 2019111003 | 2019 | 1.097 | -0.448 | 0.922 | 0.000 | 0.004 |
| 2019112003 | 2019 | 1.060 | 1.059 | 0.915 | 0.000 | 0.004 |
| 2019112005 | 2019 | 1.041 | 0.648 | 0.929 | 0.000 | 0.004 |
| 2019114003 | 2019 | 1.090 | 0.472 | 0.914 | 0.000 | 0.005 |
| 2019115005 | 2019 | 1.081 | -0.575 | 0.925 | 0.000 | 0.004 |
| 2019115002 | 2019 | 1.072 | 0.854 | 0.916 | 0.000 | 0.004 |
| 2019118001 | 2019 | 1.080 | 0.331 | 0.930 | 0.000 | 0.004 |
| 2019118002 | 2019 | 1.064 | 0.694 | 0.928 | 0.000 | 0.004 |
| 2019118005 | 2019 | 1.074 | 0.928 | 0.922 | 0.000 | 0.004 |
| 201932001 | 2019 | 0.996 | 2.305 | 0.973 | 0.000 | 0.002 |
| 201933002 | 2019 | 1.018 | 1.486 | 0.980 | 0.000 | 0.002 |
| 201933004 | 2019 | 0.995 | 1.553 | 0.972 | 0.000 | 0.002 |
| 201934003 | 2019 | 1.036 | 1.578 | 0.969 | 0.000 | 0.003 |
| 201935003 | 2019 | 1.063 | 0.525 | 0.970 | 0.000 | 0.003 |
| 201936005 | 2019 | 0.981 | 1.789 | 0.971 | 0.000 | 0.005 |
| 2019315001 | 2019 | 1.054 | 0.423 | 0.973 | 0.000 | 0.002 |
| 2019315002 | 2019 | 1.090 | -0.104 | 0.973 | 0.000 | 0.003 |
| 2019315004 | 2019 | 0.981 | 1.968 | 0.966 | 0.000 | 0.003 |
| 2019317005 | 2019 | 1.030 | 1.030 | 0.978 | 0.000 | 0.002 |

^1^PlotID: unique identifier per plot

## Alt text for figures

Figure 1. Four wheat flag leaves positioned flat on a black surface showing four different grades of glaucousness. The scale is 0: no glaucous, 1: a bit glaucous, 2: quite glaucous, 3: very glaucous.

Figure 2A. iButtons and their sun shields installed at two heights in a wheat canopy situated outdoors with day light.

Figure 2B. Infrared sensors (Arducrops) installed in wheat plots for measurement of canopy temperature. More than 10 sensors can be seen in the image in different plots in a wheat trial outdoors.

Figure 3. Graph showing albedo (dimensionless) in the Y axis and time (hours) in the X axis. Canopy albedo over the course of a day at the flowering stage during the optimal time of sowing (TOS 1) and late time of sowing (TOS 2) for the four genotypes assessed (entries 29, 21, 5 and 16). Data shown is an example of representative data from individual plots of 1 replicate for each genotype.

Figure 4. Graph showing albedo (dimensionless) in the Y axis and the X axis. Correlations of canopy albedo at noon measured with spectroradiometer ASD and albedometer across three environments: time of sowing (TOS) 1 in 2018 and 2019 and time of sowing 2 in 2018 in Narrabri field trials (NSW, Australia).

Figure 5. Graph showing albedo at noon for each entry (genotype). Canopy albedo at noon by genotype (entry number) measured by a spectroradiometer (Analytical Spectral Devices, ASD) for optimal (TOS 1) and late time of sowing (TOS 2) in 2018 and optimal sowing in 2019. Data are means ±SEM of three replicate plots.

Figure 6. Graph showing principal components (PC1 at 70.4%. and PC2 at 21.9%). Genotype by Genotype by Environment GGE biplot for Albedo in the morning (measured using hyperspectral ASD on 30 genotypes in three replicate plots) and across three environments (TOS 1 and 2 in 2018 and TOS 2 in 2019).

Figure 7. Graph showing principal components (PC1 at 70.4%. and PC2 at 21.9%). Genotype by Genotype by Environment GGE biplot for Albedo at noon (measured using hyperspectral ASD on 30 genotypes in three replicate plots) and across three environments (TOS 1 and 2 in 2018 and TOS 2 in 2019)

Figure 8. Graph showing principal components (PC1 at 70.4%. and PC2 at 21.9%). Genotype by Genotype by Environment GGE biplot for Albedo in the afternoon (measured using hyperspectral ASD on 30 genotypes in three replicate plots) and across three environments (TOS 1 and 2 in 2018 and TOS 2 in 2019).

Figure 9. Graph showing linear regression of Albedo (ASD) and light interception at noon (average value from 11 am to 1 pm) in 2018 for TOS 1 (r = 0.74, P<0.01).

Figure 10. Graph showing linear regression of Albedo (ASD) at afternoon and ΔTF (air temperature gradient between leaf and spike at flowering) at afternoon in 2018 for TOS 1 (r = 0.48, P<0.01).

Figure 11. Graphs showing regression lines for canopy temperature (X axis) and air temperature around the spike (Y axis) measured simultaneously in a plot across the season (from heading to maturity) in (A) 2018, (B) 2019.

Figure S1. Graph showing relationship for visualisation purposes of canopy albedo from spectroradiometer (ASD) (morning, noon and afternoon) (Y axis) and corresponding albedometer measurements across all repetitions, years and times of sowing (X axis).
